# Tailorable Hydrogel Fibers from High-Yield Recombinant Hagfish Intermediate Filament Proteins: A New Frontier in Biomimetic Materials

**DOI:** 10.1021/acsomega.5c13031

**Published:** 2026-05-30

**Authors:** Brianne E. Bell, Oran Wasserman, Thomas I. Harris, Hayden B. Johns, Paula E. Oliveira, Justin A. Jones

**Affiliations:** † Department of Biology, 4606 Utah State University, Logan, Utah 84322, United States; ‡ Naval Surface Warfare Center - Panama City Division, Panama City, Florida 32407, United States

## Abstract

Protein-based hydrogel fibers represent a promising class of biomaterials for biomedical applications. Previous work has demonstrated high expression yields for recombinant hagfish intermediate filament (rHIF) proteins and the ability to form rHIF-based fibers with tunable mechanical properties. Given that the natural environment of native HIF threads is aqueous, this study investigated the formation and characterization of rHIF-based hydrogel fibers in aqueous environments. Individual rHIF-α (α) and rHIF-γ_(C387S)_ (γ) proteins, as well as the 1:1 α/γ, were solubilized in formic acid (97%) at concentrations ranging from 10 to 25% w/v and spun into either deionized or saltwater coagulation baths. The resulting hydrogel fibers exhibited highly tunable mechanical properties, with elastic moduli ranging from ∼10^2^ to ∼10^3^ kPa, depending on protein concentration and coagulation conditions. Fourier transform infrared spectroscopy with attenuated total reflectance (FTIR-ATR) analysis suggests β-sheet content of 45.9–59.2% across deionized or saltwater spinning conditions, while scanning electron microscopy (SEM) revealed nanoporous structures along the hydrogel fiber axis. The combination of tunable mechanical properties, nanoporous architecture, and high recombinant protein yields, achieved using recombinant proteins alone with a green solvent and water-based coagulation, positions rHIF hydrogel fibers as a scalable, sustainably processed platform for various applications.

## Graphical abstract

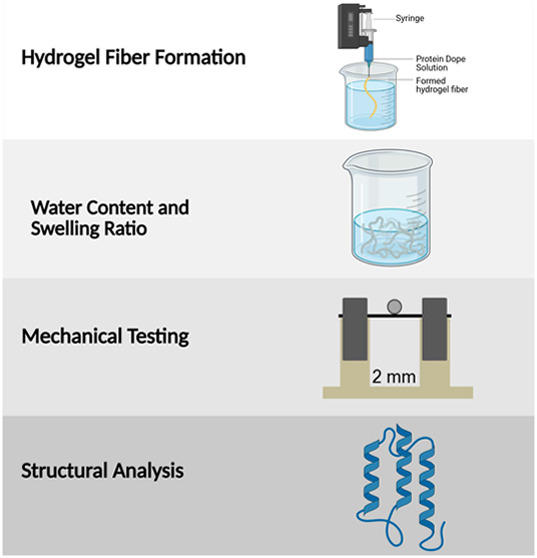

## Introduction

Protein-based hydrogels are hydrated polymer networks formed from natural or recombinant proteins.^1^ Their high water content, tunable mechanical properties, and structural similarity to the extracellular matrix make them attractive platforms for biomedical engineering, supporting cell viability, proliferation, and integration with host tissues.^1–4^ Fibrous protein hydrogels have been widely studied, including those derived from natural proteins such as collagen, elastin, and fibrin, as well as recombinant systems such as spider silk and keratin.^1,5–7^ However, many recombinant fibrous proteins face persistent challenges in large-scale production and processing.^8–10^

Intermediate filament (IF) proteins are a class of fibrous proteins with inherent mechanical robustness.^11^ Hagfish intermediate filaments (HIF), composed of α and γ IF proteins, combine strength and elasticity with biocompatibility.^12–14^ In nature, HIF form threads that are secreted extracellularly by the hagfish as part of a defensive response.^15^ The HIF threads are released into seawater, where they interact with mucin vesicles to form the defensive slime.^16–18^ Upon deployment, the threads function in a fully hydrated state, with the intermediate filaments remaining hydrated throughout their defensive role.^19^ This natural deployment in water suggests an intrinsic compatibility with hydrated, hydrogel-like environments.

HIF proteins extracted from hagfish slime have been utilized to form hydrogel fibers and films cross-linked by biocompatible divalent ions (Mg^2+^, Ca^2+^), with stiffness tuned by cross-link density.^20–23^ Ionic cross-linking approaches are generally preferred over chemical cross-linking, as chemical cross-linkers such as glutaraldehyde are often toxic and require postprocessing removal.^24–26^ The use of water-based coagulation baths avoids the need for toxic organic solvents commonly used in other fiber-spinning approaches. In addition, HIF proteins have been solubilized in formic acid in these systems, which is classified as a green solvent due to its biodegradability and relatively low environmental impact.^27^ Together, these processing choices position HIF proteins as a promising candidate for sustainably processed, protein-based hydrogel materials.^21,28^

Our previous work demonstrated the ability to express and recover recombinant hagfish intermediate filament (rHIF) proteins at high expression yields (>8 g of purified dry protein per liter of bioreactor media) using a scalable filtration-based purification process.^29,30^ Recombinant protein yield has been identified as a primary factor in sustainable protein-based biomaterial production.^31^ In addition, we have demonstrated the ability to form rHIF-based dry fibers with robust and tunable mechanical properties for full-length rHIF proteins and rHIF protein constructs.^29,30,32^ The high expression yield exceeds that of previously reported full-length or even larger recombinant fibrous proteins,^33–36^ reducing a major barrier to presenting rHIF-based biomaterials as a viable option for industrial applications. Collectively, the high expression yield, formic acid solubilization, and water-based fiber formation establish a sustainable processing framework for rHIF hydrogel fibers.

In this study, we investigate hydrogel fibers formed from rHIF-α (α), rHIF-γ_(C387S)_ (hereafter γ), and their natural 1:1 α/γ ratio.^37^ Protein concentrations, spinning parameters, and testing conditions were varied to establish structure–property relationships. Water content and swelling ratios were measured and benchmarked against other protein hydrogels. Selected fibers were analyzed by FTIR-ATR and computational predictions (AlphaFold3) (Figure S1), and fiber morphology was examined by SEM. This thorough characterization provides a framework for tailoring rHIF hydrogel fibers to application-specific mechanical and functional requirements.

## 2 Materials and Methods

### 2.1 Hydrogel Fiber Formation

Hydrogel fibers were prepared by solubilizing rHIF proteins in formic acid and extruding them into saltwater (SW) or deionized water (DW) baths. The purified recombinant proteins, α and γ, were obtained using previously described methods and were solubilized as described by Bell et al. and Wasserman et al.^30,32^ The γ variant (rHIF-γ_(C387S)_) was chosen over the native sequence because of its proven ability to express and purify at large scale, with disulfide cross-links eliminated during those stages.^29,30^ The dopes were made with 2–3 mL of 97% formic acid (FA) (Aesar A13285) in a 4 mL glass vial (Figure 1, Step 1) at protein concentrations of 10%, 15%, 20%, and 25% w/v, with the proteins in the 1:1 α/γ being added at equal proportions. The 1:1 α/γ dope was chosen because it reflects the protein combination seen in naturally occurring HIF.^21,38^ After the dopes were allowed to solubilize overnight (Figure 1, Step 2), under constant rotation, the dope was transferred from the 4 mL glass vial to 1.5 mL centrifuge tubes and centrifuged for 15 min at 18,000 rcf (Figure 1, Step 3). The solubilized and centrifuged dopes were placed into a 3 mL syringe fitted with a 26 G, 25.4 mm blunt-tip needle and loaded onto a custom spinning machine.^39,30,32^ Although this device was previously reported to produce dry fibers with applied stretch, this study only used the extruder and coagulation bath of the system (Figure 1, Step 4), with the extrusion rate kept around 2 mL h^–1^. The coagulation bath had two conditions: saltwater (SW, Instant Ocean Marine Fast Dissolving Sea Salt at 36 g/L) and deionized water (DW). After spinning was complete, the hydrogel fibers were transferred to beakers containing fresh water (Figure 1, Step 5), SW or DW, to avoid degradation caused by FA that leached into the coagulation solution. These processes resulted in four experimental conditions: SW-SW, SW-DW, DW-SW, and DW-DW, where the first water designation is the coagulation bath, and the second is the testing water.

**Figure 1:**
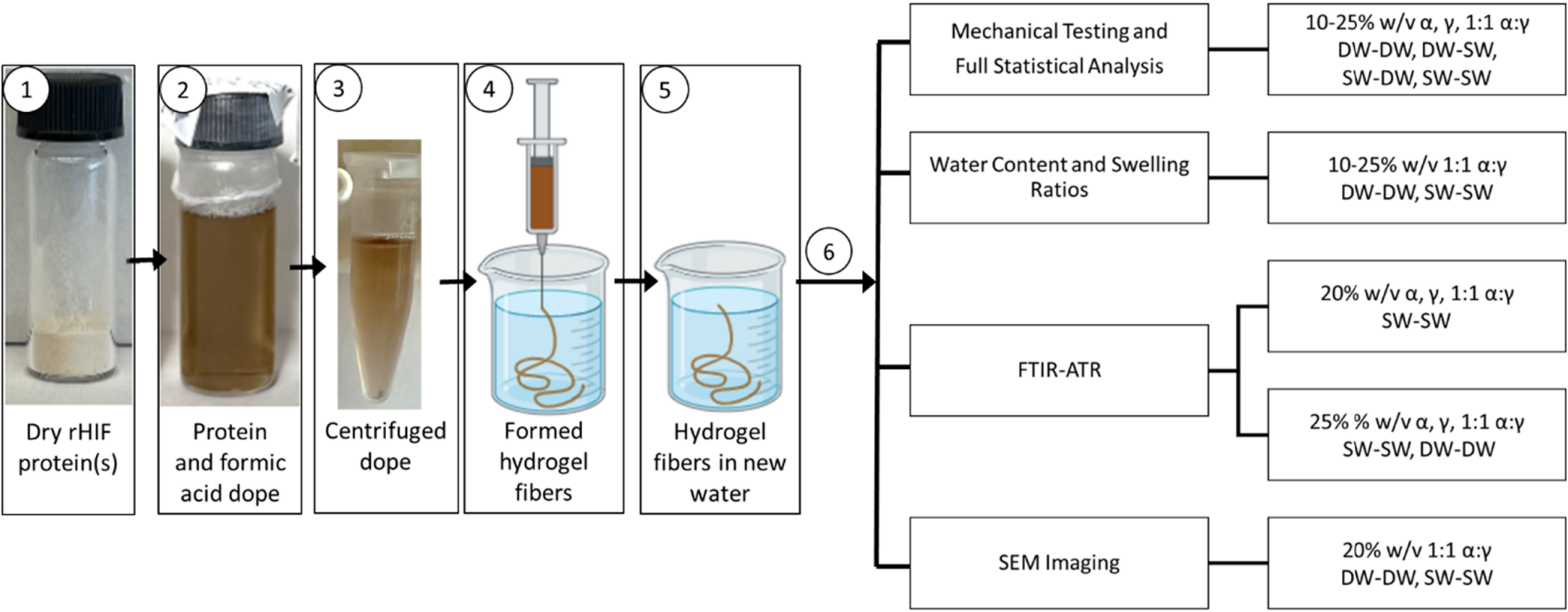
Experimental process map overview with numbered steps (1–6) for the hydrogel fiber formation process. (1) Representative images of the dried protein, (2) solubilized protein dope, and (3) centrifuged dope. Followed by BioRender icons depicting (4) hydrogel fiber formation and (5) fiber storage. The final portion of this figure 6) depicts the experiments performed on the hydrogel fibers and the fiber types used.

### 2.2 Water Content and Swelling Ratio Determinations

To determine the water weight percentage of the hydrogel fibers (eq 1), a 3 mL dope was created at protein concentrations of 10%, 15%, 20%, and 25% w/v using the 1:1 α/γ formulation. 1.5 mL from each dope (half of the total dope solution volume) was spun into DW and the other half into SW. The hydrogel fibers, SW- and DW-spun, were rinsed in clean, unused DW to remove formic acid and salt prior to proceeding to the next steps. The hydrogel fibers were removed from the water as a clump, placed on a glass surface, and cut into roughly 1.0 × 0.5 cm^2^ sections. These sections were dabbed dry with Kimwipes until no watermarks remained, and the hydrated mass (*m*_hydrated_) was recorded. The hydrogel fiber bundles were then frozen at −20 °C while suspended in DW and lyophilized on a Harvest Right Freeze-Dryer, using the method described by Bell et al.^30^ The fully dried hydrogel fiber groups (*m*_lyophilized_) were then weighed, and the percent water weight (% water) was calculated (eq 1):

1 $$\%\;water=\frac{m_{hydrated}-m_{lyophilized}}{m_{hydrated}}\times 100\%$$

Additionally, the mass swelling ratio or absorption capacity (*Q*_m_) was determined using the same measured masses and following the equation used by Tolentino et al.^40^ (eq 2):

2 $$Q_{m}=\frac{m_{hydrated}-m_{lyophilized}}{m_{lyophilized}}\times 100\%$$

In addition to comparing the lyophilized and hydrated masses, the diameters were also measured in the lyophilized and hydrated states to determine the swelling ratio as it relates to the diameter of the fixed-length fibers. The diameters of lyophilized hydrogel fibers were measured using a Motic BA310 microscope at a total magnification of 400X, and measurements were obtained with Motic Image Plus software (v. 3.1).^29,30,32^ Five fibers were measured in their dried state for each group. Each fiber sample had one image with three measurements per image, resulting in 15 total measurements per fiber type. The hydrated hydrogel fiber diameters were measured alongside mechanical testing through the “Analyze and Review Images” option in CellScale’s MicroTester software (v. 6.08).^41^ The crosshairs were used to measure the diameter of the hydrogel fiber at the start of each test, using three measurements per 2 mm-long fiber segment. The average diameter measures were used to calculate the diameter swelling ratio (*Q*_d_) (eq 3):

3 $$Q_{d}=\frac{d_{\mathrm{hydrated}}-d_{\mathrm{lyophilized}}}{d_{\mathrm{lyophilized}}}\times 100$$

### 2.3 Mechanical Testing

Mechanical performance of hydrated rHIF hydrogel fibers was characterized using a submerged three-point bending test. All the hydrogel fibers were tested using a CellScale MicroTester G2 (CellScale Biomaterials Testing) utilizing either a 0.4064 mm or 0.5588 mm diameter tungsten beam, the included bath, and a custom-built 2 mm spacer (Figures 2,3, S2).^41^ The custom spacer had a 2 mm gap, with the support towers (gray checkerboard-filled U-shape in Figure 3) high enough to cover the device’s extension limit while allowing the test fiber to be submerged. The bath was filled with either SW or DW, matching the second portion of the bath label after the dash. Individual hydrogel fibers (black lines in Figure 3) were spread across the mounted spacer gap, ensuring the thread was visibly taut but not stretched (Figure 2A). This ensured an accurate and reproducible testing procedure, as the absence of pretest stretching enabled consistent measurement. Custom clamps^41^ (Figure S3) were then placed on top of each mounting tower to secure the hydrogel fiber. The beam was set in place so that it reached over the thread by about 1 mm and rested on top of it (circle in Figures 2 and 3). As the test progressed, the beam moved downward (Figure 2, A–C),3 at a constant rate of 0.0167 mm s^–1^, with displacement and applied force recorded by the MicroTester software (v. 6.08).

**Figure 2:**
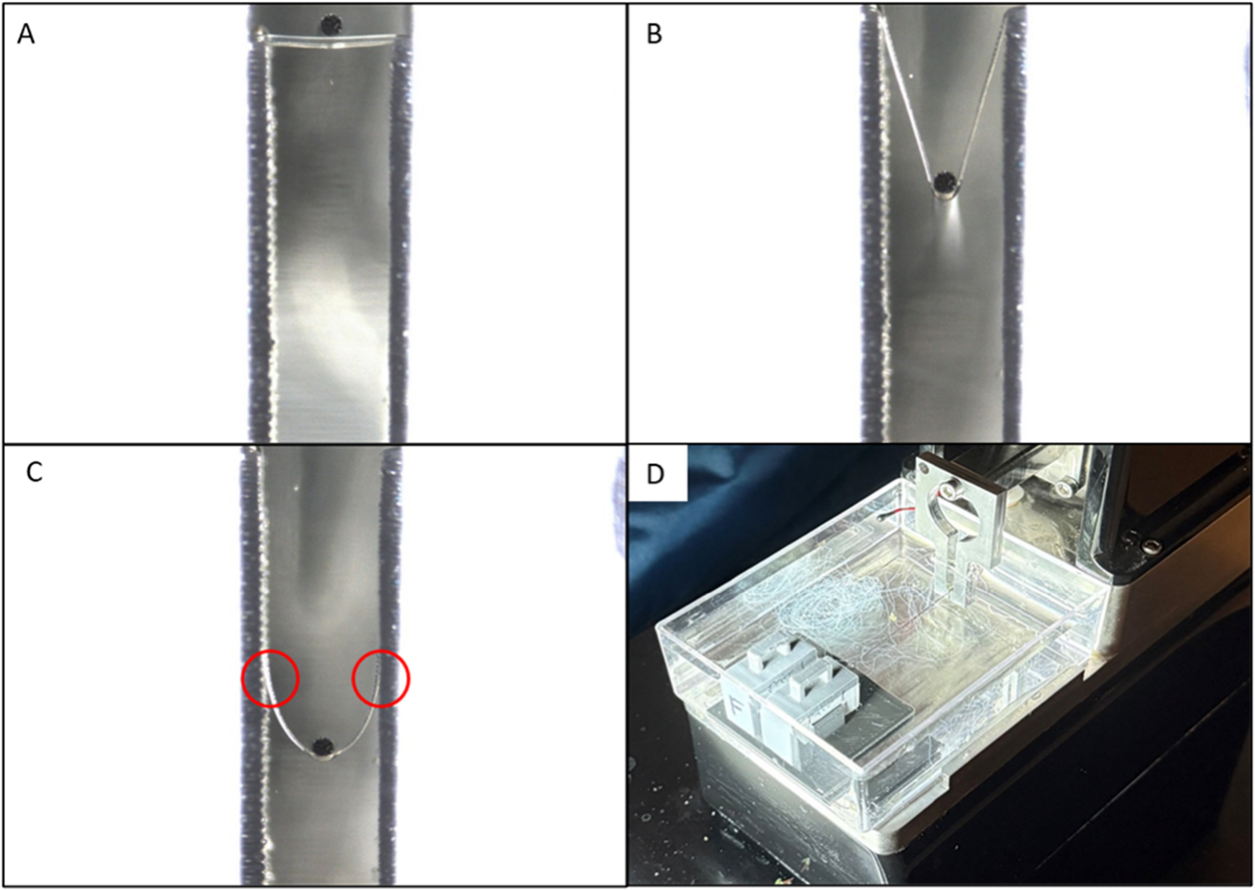
Representative hydrogel fiber mechanical testing progression. (A) hydrogel fibers mounted on the support towers prior to the mechanical testing. (B) A representative time point during mechanical testing, showing an approximate triangular shape of the hydrogel fiber. (C) Visual break (indicated by the red circles) in the hydrogel fiber, indicating the end of the test. (D) CellScale MicroTester G2 testing setup. Pictures A–C were taken via the MicroTester software v. 6.08.

**Figure 3:**
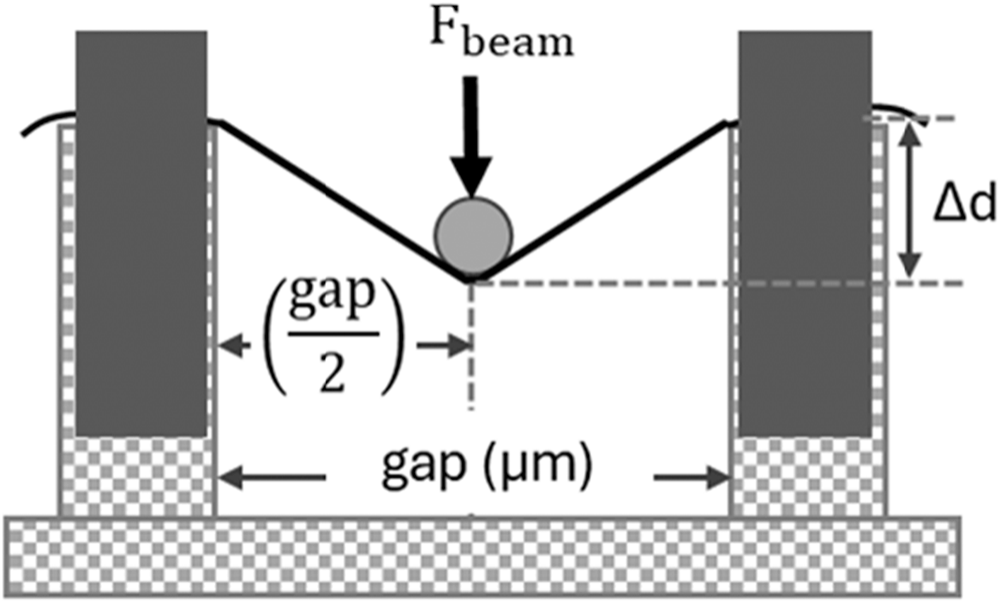
Direct inline diagram of the custom hydrogel fiber securing device with custom clamps (gray) securing a hydrogel fiber (black line) across the spacer (gray checkerboard U-shape). The necessary measures (vertical displacement, gap, and force) shown in the Equations are included in the diagram.

The software (MicroTester software v. 6.08) with the compression template (Basic Compression) was then adjusted to the correct beam diameter, length, and modulus. The tests were run for 600 s without recovery, using a compression magnitude of 10 mm and a collection frequency of 1 Hz, until the hydrogel fiber broke or the test parameters were completed (Figure 2C). A representative rHIF hydrogel fiber mounted and going through testing is shown in Figure 2, A–C, with the full testing setup depicted in Figure 2, D.

The test data were exported to an Excel spreadsheet where the raw force load (μN) and raw tip displacement (μm) were combined with the measured hydrated diameter to calculate stress, strain, energy to break, and elastic modulus. Since the test used was a three-point tension test, adjustments to stress and strain were made to account for the different geometry compared with traditional tensile testing (eqs 4–7).

The strain (ε) was calculated using eq 4:

4 $$\varepsilon =\frac{\sqrt{{\left(\Delta d\right)}^{2}+{\left(\frac{\mathrm{gap}}{2}\right)}^{2}}-\left(\frac{\mathrm{gap}}{2}\right)}{\frac{\mathrm{gap}}{2}}$$

where Δ*d* denotes the vertical displacement of the beam (μm), and “gap” refers to the width (μm) of the gap between the support towers (Figure 3).

The resolved stress (σ) (eq 7) calculation involved first determining the angle between the hydrogel fiber and the beam’s vertical displacement (χ) (eq 5):

5 $$\chi =\tan^{-1}\left(\frac{\Delta d}{\frac{\mathrm{gap}}{2}}\right)\times \frac{180^{{}^{\circ}}}{\pi }$$

Next, the stress acting directly on the fiber (σ_fiber_) needed to be determined using the applied force and diameter measurements (eq 6):

6 $$\sigma_{\mathrm{fiber}}=\frac{F_{beam}}{\pi {\left(\frac{diameter}{2}\right)}^{2}}$$

Here, the diameter measurement is the average diameter of the hydrogel fiber (μm) and *F*_beam_ is the force from the beam acting on the hydrogel fiber (μN). Finally, the resolved stress σ is given by

7 $$\sigma =\sigma_{\mathrm{fiber}}\sin\left(\chi \frac{\pi }{180^{{}^{\circ}}}\right)$$

The term “resolved stress” has been abbreviated to “stress” in the Results for simplicity. The energy to break the hydrogel fiber was determined by calculating the area under the stress–strain curve.^42^

The mechanical performance of the hydrogel fibers at different concentrations and conditions (Table S3, Figure S5) dictated the selection of the parameters for each experimental characterization procedure. Each experimental procedure section that follows contains an explanation of what mechanical characteristic was the key driver for specific consideration. Figure 1, in addition to providing a schematic for the process of going from purified protein powder to hydrogel fibers, shows which dope formulations and water types were used in further characterization tests. Table S1 provides a comprehensive list of the dopes/constructs used to form hydrogel fibers in this experiment, organized by the experimental characterization method.

### 2.4 Structural Analysis and Modeling

To investigate structural features underlying fiber performance, selected rHIF hydrogel fibers were analyzed using FTIR-ATR and then compared to AlphaFold3-predicted protein structures. The selected hydrogel fibers for FTIR-ATR analysis were 20% SW-SW, 25% SW-SW, and 25% DW-DW for each protein and their 1:1 combination (Table S1, Figure 1). These conditions and concentrations were chosen for FTIR-ATR analysis because saltwater conditions demonstrated more consistent mechanical properties across constructs, while the 25% DW-DW condition exhibited the highest mechanical properties among DW-DW hydrogel fibers and served as a comparison to the SW-SW hydrogel fibers. Additionally, these conditions were selected to enable comparisons with previously reported stretched dry fibers from our group.^32^

The hydrogel fibers were sent to Arizona State University’s Eyring Materials Center Facilities, where they were processed using a PerkinElmer Frontier FTIR with a Globar source, utilizing 50 scans and the Pike diamond ATR module. The hydrogel fibers were dried via a tissue wipe prior to being pressed onto the Diamond ATR to reduce background interference. The raw data were deconvoluted using OriginPro 2023 (OriginLab) software after the absorbance data were normalized to the maximum of the Amide I peak.^20^ Deconvolution of the Amide I region (∼1550–1720 cm^–1^) followed similar procedures to those described previously.^20,29,32,43^ A straight-line baseline correction was applied prior to implementing a Savitzky-Golay smoothing method with a second-order polynomial and 5–7 points of the window to find absorbance peaks.^32^ Absorbance locations were selected based on the results of peak fitting, the minima of the second derivative around 1650 and 1680 cm^–1^, and fit parameters of 400 iterations and a tolerance of 1 exp^–14^ that resulted in the highest *R* ^2^ value.^29,32^

The individual α and γ proteins and the 1:1 α/γ combination were modeled using AF3 (Figure S1), a deep learning-based model capable of predicting the three-dimensional structures of biomolecular complexes, including proteins, nucleic acids, and small molecules.^44^ Predictions were ranked using a combination of predicted template matching (pTM) and interface predicted template matching (ipTM) scores, which measure the accuracy of the predicted structure.^45,46^ Confidence estimates for the predictions were obtained from the per-residue predicted local distance difference test (pLDDT) scores.

### 2.5 SEM Imaging

The morphology of rHIF hydrogel fibers was examined with scanning electron microscopy (SEM). Cross-sectional and longitudinal images were used to identify pore structure and surface features produced under different coagulation conditions. Imaging was performed on the 20% 1:1 α/γ SW-SW and 20% 1:1 α/γ DW-DW samples to demonstrate visible differences and similarities between hydrogel fibers spun in SW and those spun in DW. These images were captured using a Quanta SEM (FEI, Quanta FEG 650 SEM) with xT Microscope Control software. Images of the length of individual hydrogel fibers were captured using 1000× magnification in the environmental mode (E-SEM) in a chamber of 3.60 Torr (∼480 Pa), 1 °C, and imaged at a dwell time of 15 μs after mounting dried single hydrogel fibers to a sample mount with double-sided copper tape. Images of the cross-section of the hydrogel fibers were obtained at 5000X and 10,000× magnification after drying and placing the cut hydrogel fibers vertically onto a separate sample holder capable of mounting samples on the side. The cross sections were imaged in a low-vacuum mode SEM with the chamber at 0.376 Torr (∼50 Pa) and imaged at a dwell time of 15 μs. The change in vacuum modes was made because the images were taken at different orientations, angles, and views of the rHIF hydrogel fibers. The pore diameters of the cross-sectional micrographs of the hydrogel fibers were measured using ImageJ.

### 2.6 Statistical Analysis

Statistical analyses were performed to evaluate differences in swelling behavior, water content, diameter, and mechanical properties across the experimental groups. For each swelling ratio, water content, and diameter measurement, Pearson’s correlation coefficients were calculated and plotted in a correlation plot (Figure S4) to help determine relationships of fiber properties based on coagulation bath water type. The mechanical data are presented as mean ± standard deviation (Tables 1 and S3). One-way ANOVA with posthoc Tukey tests were performed on the mechanical data using R software. The statistical significance was set at α = 0.05.

**Table 1:** Summary of Mechanical and Structural Properties of a Selection of Hydrogel Fibers, with *n* Being the Number of Tests Performed^a^

% w/v protein, water types	*n*	stress (MPa)	strain (mm mm^–1^)	energy to break (MJ m^–3^)	elastic modulus (kPa)	% β-sheet	% α random coil/helices
α							
20% SW-SW	11	1.43 ± 0.21 [B]	5.99 ± 0.32 [B]	4.64 ± 0.45[B]	436.8 ± 75.8 [B]	59.2	40.8
25% SW-SW	11	2.13 ± 0.39 [A]	7.35 ± 2.13 [A]	10.2 ± 4.16 [A]	763.5 ± 105 [A]	50.5	49.5
25% DW-DW	15	1.04 ± 0.13 [C]	2.87 ± 0.23 [C]	1.47 ± 0.20 [C]	403.7 ± 130 [B]	46.1	53.9
1:1 α/γ							
20% SW-SW	12	1.38 ± 0.33 [B]	4.42 ± 1.26 [A]	3.35 ± 1.81 [B]	589.5 ± 168 [B]	54.2	45.8
25% SW-SW	14	2.24 ± 0.32 [A]	4.97 ± 1.00 [A]	6.20 ± 2.19 [A]	971.2 ± 102 [A]	52.2	47.8
25% DW-DW	12	1.60 ± 0.40 [B]	2.47 ± 0.23 [B]	2.01 ± 0.48 [B]	1041 ± 294 [A]	45.9	54.1
γ							
20% SW-SW	12	1.33 ± 0.30 [A]	4.73 ± 1.13 [A]	3.28 ± 0.48 [A]	502.9 ± 91.6 [A]	47.8	52.2
25% SW-SW	11	1.18 ± 0.13 [A]	4.05 ± 0.41 [A]	2.54 ± 0.46 [B]	426.3 ± 49.4 [A, B]	59.0	41.0
25% DW-DW	11	0.85 ± 0.13 [B]	2.74 ± 0.16 [B]	1.21 ± 0.18 [C]	382.9 ± 74.8 [B]	50.3	49.7

a The bracketed letters indicate the ANOVA-Tukey group assignments for this selection of tests

## 3 Results

### 3.1 Hydrogel Fiber Formation, Water Content, and Swelling Ratios

All dopes were made in FA and successfully spun as described previously, with hydrogel fibers forming in the coagulation bath and removed to fresh baths for testing (Figure 1).^30,32^ The hydrogel fibers (10, 15, 20, and 25% w/v 1:1 α/γ), whether formed in SW or DW, demonstrate high water content (eq 1; Figure 4, B; Table S2) and absorption capacity or mass swelling ratios (Figure 4, D). The DW-formed hydrogel fibers demonstrate a high water content that decreases from 81% to 75% as the concentration increases from 10% to 20% w/v, with 25% w/v having the same water content as 20% w/v for the 1:1 α/γ DW hydrogel fibers. The SW-formed hydrogel fibers have a lower water content, which decreased from 75% to 66% water, while the protein concentration of the 1:1 α/γ hydrogel fibers increases from 10 to 20% w/v. The water content increases negligibly from 66% in the 20% w/v fibers to 68% in the 25% w/v hydrogel fibers. The DW formed hydrogel fibers have a strong negative correlation coefficient (−0.96) between their hydrated diameters and their water content, while the SW-formed hydrogel fibers have a slightly weaker correlation coefficient (−0.79) for the same comparison.

**Figure 4:**
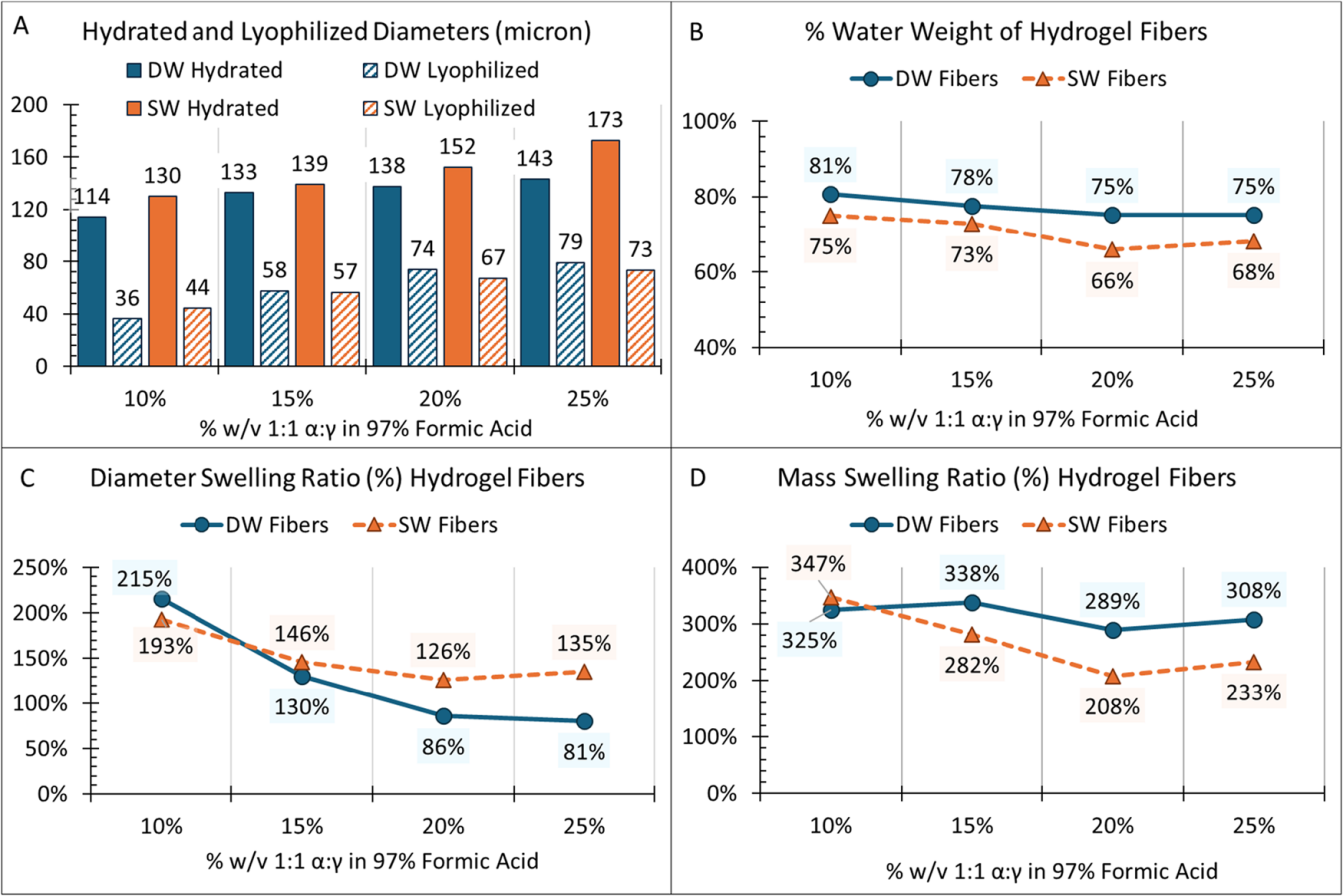
Diameters, water content, and swelling ratios of 1:1 α/γ DW-DW and SW-DW hydrogel fibers at protein concentrations of 10%, 15%, 20%, and 25% w/v. (A) Hydrated and lyophilized diameters, (B) water content by mass, (C) diameter swelling ratio, and (D) mass swelling ratio.

The DW-formed hydrogel fibers generally have a higher mass swelling ratio (eq 2; Figure 4, D; Table S2) than the SW-formed hydrogel fibers, except with 10% 1:1 α/γ concentration, where the SW is higher (347% vs 325%). The swelling ratios of the DW hydrogel fibers increase slightly (325% to 338%) as the concentration increases from 10% to 15% w/v, then decrease to a low of 289% at 20% w/v. The swelling ratio then increases slightly to 308% for the 25% w/v DW. The SW-formed hydrogels follow a simpler trend where the mass swelling ratio decreases from 347% to 208% as the concentration increases from 10% to 20% w/v, then increases slightly to 233% at 25% w/v. The DW formed hydrogel fibers have a weak negative correlation coefficient (−0.48), while the SW-formed hydrogel fibers have a stronger negative correlation coefficient (−0.78) between their hydrated diameters and their mass swelling ratios.

The diameter swelling ratio (eq 3; Figure 4, A, C; Table S2) showed similar patterns for both DW- and SW-formed hydrogel fibers, with SW generally having slightly higher values than DW, except for the 10% w/v hydrogel fibers, where DW was higher. The DW hydrogel fibers had a maximum diameter swelling ratio of 215% for the 10% w/v hydrogel fibers, decreasing to 81% for the 25% w/v hydrogel fibers. This decrease is accompanied by an overall strong negative correlation (−0.99) between the wet diameter and the diameter swelling ratio of the DW hydrogel fibers. The SW-formed hydrogel fibers showed a less drastic decrease in their diameter swelling ratio, decreasing from 193% to 135% as the concentration increased from 10% to 25% w/v. This trend agrees well with the overall Pearson’s correlation coefficient between the hydrated diameter and the diameter swelling ratio (−0.73).

### 3.2 Mechanical Performance and Secondary Structure of rHIF Hydrogel Fibers

Mechanical properties varied markedly with protein composition, concentration, and environmental conditions (Figure 5, A–D, Table 1). For α fibers, increasing protein content from 20% to 25% in SW–SW significantly enhanced tensile stress (1.43 → 2.13 MPa), strain (5.99 → 7.35 mm mm^–1^), and energy to break (4.64 → 10.2 MJ m^–3^), accompanied by a higher elastic modulus (437 → 764 kPa). These gains were largely lost for DW–DW conditions, where stress, strain, and energy to break dropped to their lowest α-fiber values, although modulus returned to near the 20% SW–SW level.

**Figure 5:**
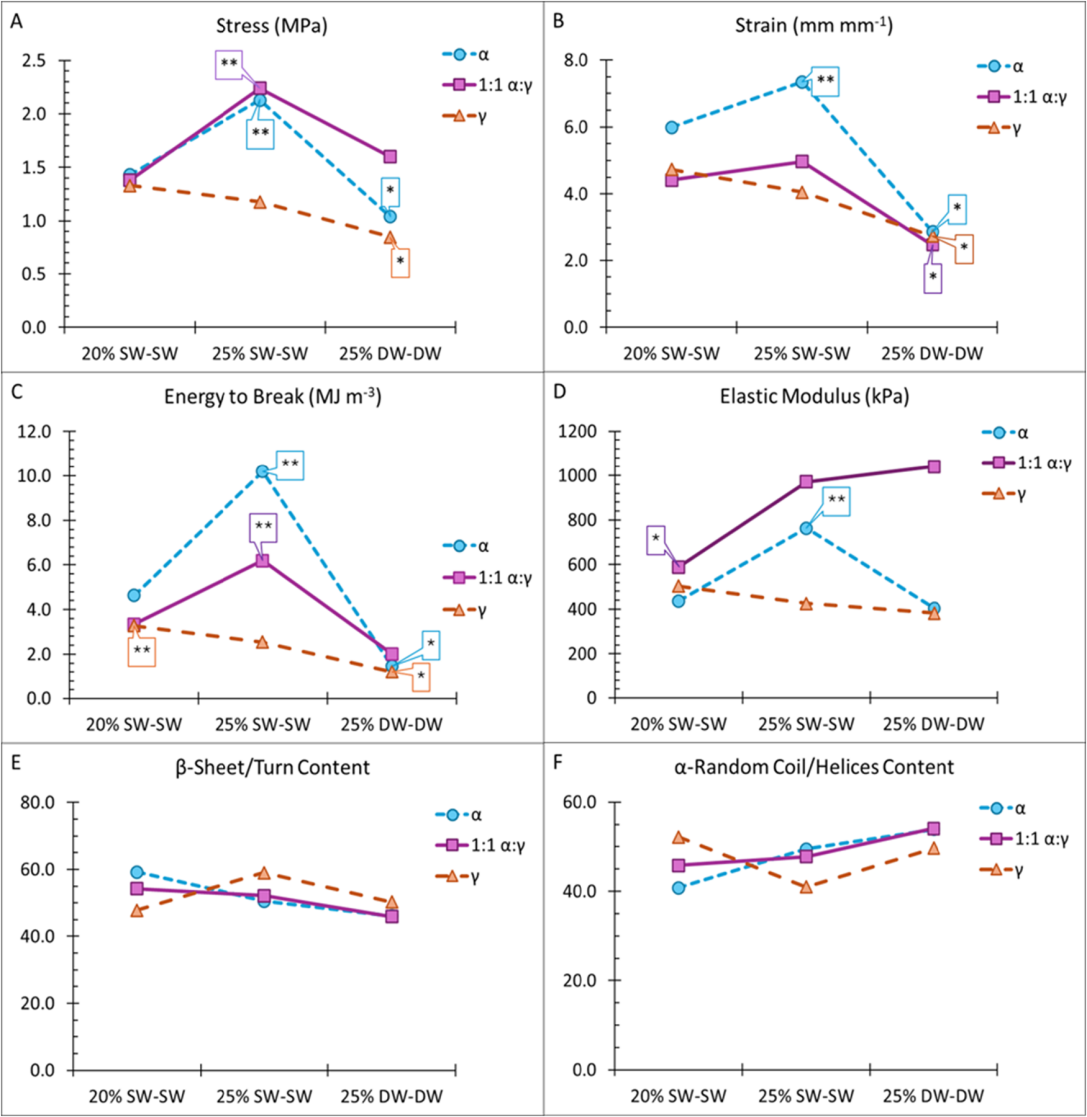
Visual trends of the mechanical (A–D) and structural (E, F) characteristics for the selective hydrogel fibers. (A) mechanical stress in MPa, (B) ultimate strain in mm mm^–1^, (C) energy to break in MJ m^–3^, (D) elastic modulus in kPa, (E) FTIR-derived β-sheet content approximations, and (F) α-random coil/helices content for selected hydrogel fibers. Values reflect conventional FTIR deconvolution and may overestimate β-sheet due to spectral overlap with coiled-coil helices. Asterisks signify significantly different property values within that protein group.

The 1:1 α/γ blends showed a similar concentration-dependent increase in stress (1.38 → 2.24 MPa) and modulus (590 → 971 kPa) in SW–SW, but unlike α alone, the modulus continued to increase for DW–DW conditions (1041 kPa) despite reduced strain and energy to break.

In contrast, γ fibers exhibited little benefit from a higher concentration in SW–SW, with stress, strain, and energy to break either unchanged or slightly reduced. All mechanical metrics declined further for DW–DW conditions, with stress and energy to break reaching the lowest values among all compositions (0.85 MPa and 1.21 MJ m^–3^, respectively).

Across all compositions, energy to break mirrored the combined stress–strain trends: α fibers at 25% SW–SW were the most damage-tolerant, while all compositions converged to similarly low energy to break (1–2 MJ m^–3^) for DW–DW conditions.

Secondary structure was examined using FTIR deconvolution, with representative values shown in Figure 5, E,F, and Table 1. Across compositions and processing conditions, only modest differences were observed in β-sheet/turn and α-random coil/helical content. To place these findings in context, we also compared them to AlphaFold3 (AF3) structural predictions (Figure S1), which indicate a predominantly α-helical coiled-coil architecture. This comparison highlights that the 45–60% β-sheet values estimated by FTIR should be interpreted cautiously, as coiled-coils produce amide I bands near 1628–1640 cm^–1^ that can be misassigned as β-sheets.^47^

### 3.3 SEM Imaging

The surface of the hydrogel fibers at 1,000× magnification showed that the observable texture of the hydrogel fibers in DW and SW is very similar (Figure 6, A,B). Both hydrogel fiber sets exhibit a smooth external surface without visible pores at this magnification. At 5,000× magnification (Figure 6, C,D), the cross-sectional surfaces of both hydrogel fiber types appear rough, likely due to razor-blade cutting, with some porous structures visible within the hydrogel fiber matrix. Upon looking at the cross-section of the hydrogel fibers at 10,000× magnification (Figure 6, E,F), the rHIF hydrogel fibers exhibited submicron pore features within their structure that appear to go across the hydrogel fiber axis. These pores appear to be smaller than 1 μm in diameter in both SW- and DW-spun hydrogel fibers, indicating that they are submicron or nanosized.^48^

**Figure 6:**
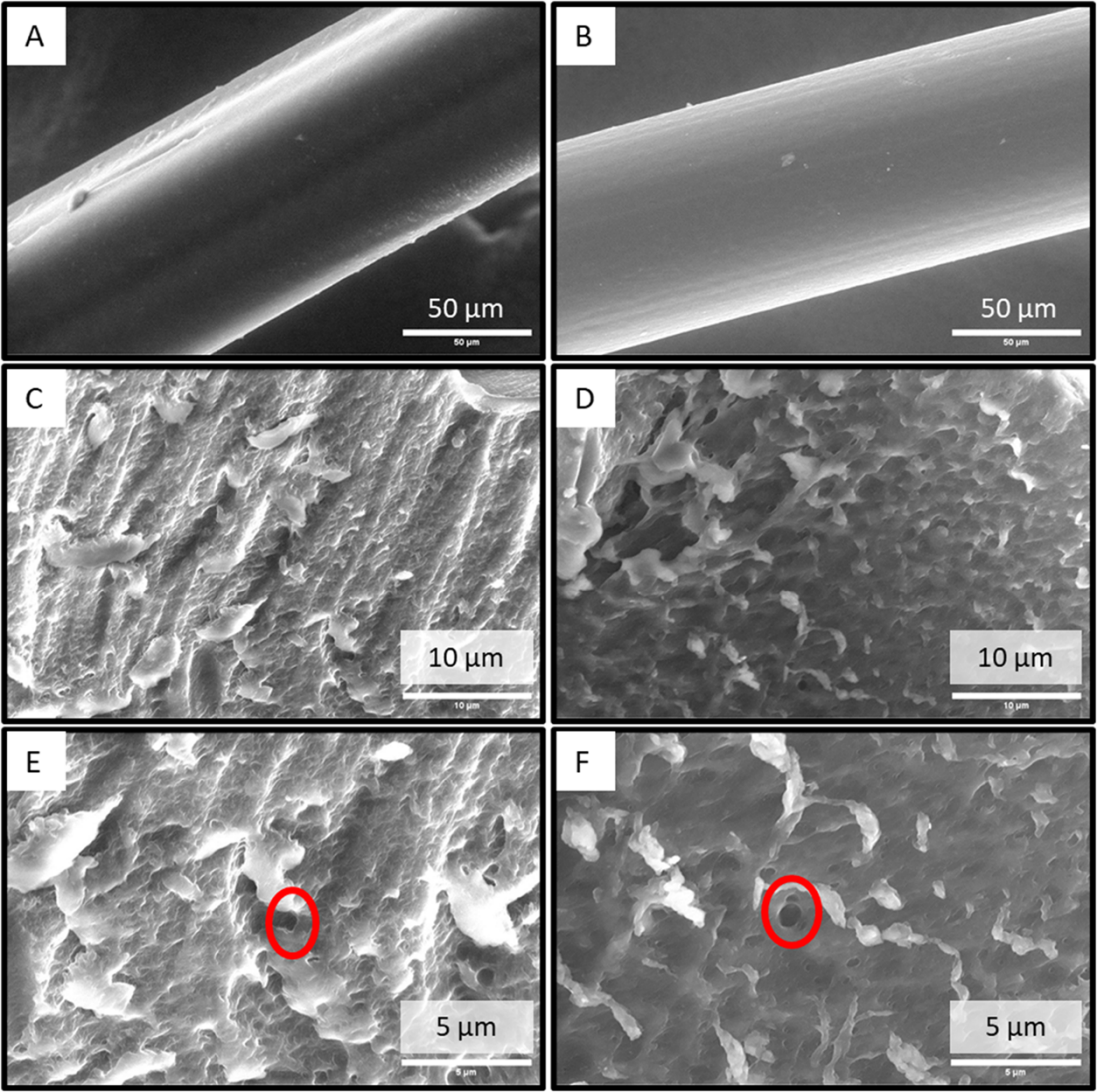
E-SEM images of the surface and cross-sectional views of hydrogel fibers, 20% 1:1 α/γ DW-DW on the left and 20% 1:1 α/γ SW-SW on the right. (A, B) Surface SEM images of the hydrogel fibers at 1000× magnification with a 50 μm scale bar. (C, D) SEM images of the cross-section of the hydrogel fibers at 5000× magnification with a 10 μm scale bar. (E, F) SEM images of the cross-section of the hydrogel fibers at 10,000× magnification with a 5 μm scale bar. Red circles (E, F) highlight the observed nanopore structures.

## 4 Discussion

The rHIF hydrogel fibers retain 66–81% water by mass, with the DW typically having higher water content than the SW fibers (Figure 4), likely due to the cross-linking by the divalent ions Mg^2+^ and Ca^2+^, in the artificial ocean water.^20–23^ This water retention range is comparable to that of collagen (>99.5%), fibrin (>98%), and silk fibroin (>90%).^49–51^ The relationship between cross-linking density and water content is complex: higher cross-linker concentrations reduce swelling ratios by decreasing mesh size, though this also diminishes ionic conductivity.^52^ The presence of multivalent ions substantially decreases swelling capacity and water uptake in polyelectrolyte hydrogels, with Al^3+^ forming mechanically stiff barrier layers that further restrict fluid absorption.^53^ Processing conditions and material composition significantly influence water content and swelling behavior across hydrogel systems,^54,55^ though the values for rHIF hydrogel fibers fall within ranges reported for established protein-based biomaterials.^49–51^ The rHIF hydrogel fiber platform, which combines high recombinant protein yields with the use of a green solvent and water-based coagulation, supports its potential as an innovative, scalable, and sustainably processed biomaterial for various applications. Prior work has identified an expression yield of 10 g/L using immobilized metal affinity chromatography as a benchmark for economically viable recombinant fibrous protein production.^56^ The rHIF expression yield exceeding 8 g/L,^30^ combined with our previously reported tangential flow filtration purification scheme that avoids the cost of affinity chromatography,^31^ approaches this economic threshold. Future work will more precisely quantify production costs to assess the economic viability of rHIF-based biomaterial production at scale.

The tunable elastic modulus range (106–1041 kPa) observed for rHIF hydrogel fibers opens possibilities for applications across diverse fields requiring specific mechanical properties (Tables 1, S3). For tissue engineering applications, hydrogels require elastic moduli that approximate those of target biological tissues.^5^ Several human tissues exhibit elastic moduli ranging from approximately 100 to 1200 kPa,^57–59^ which these rHIF hydrogel fibers encompass most of this spectrum. Beyond tissue engineering, the observed mechanical tunability positions rHIF hydrogel fibers for further investigation as a suitable biomaterial for applications such as 3D bioprinting and biosensors.^60,61^ rHIF proteins, along with the production and processing methodologies developed in our laboratory,^29,30,32^ offer advantages over other hydrogel systems that require complex chemistries, such as photodegradable cross-links, pH-responsive polymers, or redox-controlled switches.^62–64^ Recent biomass-derived hybrid hydrogels combining polysaccharides, such as carboxymethyl chitosan, with silk sericin or cellulose-based polymers have demonstrated tunable mechanical properties for wound healing, electronic skin, and integrated bioelectronic applications.^65,66^ In contrast to these multicomponent hybrid systems, rHIF-based hydrogel fibers achieve a broad range of tunable mechanical properties from rHIF proteins alone. While these mechanical and structural properties support the broad application potential of rHIF hydrogel fibers, future evaluations of cytotoxicity and biocompatibility will be needed to confirm their suitability for biomedical and bioelectronic applications.

FTIR-ATR and computational modeling provided insight into the structural basis for the observed mechanical tunability. FTIR-ATR of hydrogel fibers indicated high β-sheet content across formulations (45.9–59.2%), with complementary decreases in α/random coil content. However, caution is warranted: coiled-coil α-helices in intermediate filament proteins can produce amide I bands near 1628–1640 cm^–1^ that are often misassigned as β-sheet structures.^47^ Consistent with this interpretation, AlphaFold3 (AF3) modeling predicted predominantly α-helical coiled-coil architectures for rHIF α and γ proteins. Thus, the apparent β-sheet content from FTIR analysis likely represents a combination of true β-sheets, induced in part by formic acid processing,^67^ and misassigned coiled-coil signal. This interpretation is further supported by comparisons to rHIF dry fibers spun from HFIP, which exhibited lower β-sheet content (9–14%),^29^ as well as trends in silk fibroin systems where aqueous environments enhance β-sheet formation.^68^ Together, these results suggest that both processing conditions and hydration state influence the secondary structure of rHIF fibers, which in turn impacts their mechanical performance. For a more comprehensive look at the rHIF hydrogel fibers’ structural composition, further studies like X-ray diffraction would be beneficial. While the central core domains of HIF proteins in the natural system are known to assemble into α-helical coiled-coil heterodimers,^38^ future structural studies employing techniques such as X-ray diffraction and cryo-electron microscopy would be needed to determine whether the coiled-coil conformation is present in the rHIF hydrogel fibers processed in this study.^38,69^

SEM imaging revealed morphological differences linked to spinning conditions. Cross sections showed denser cores in DW–DW fibers, with both types exhibiting nanoporous structures (<1 μm pores) spanning the fiber axis. The submicron pore size likely prevents cellular infiltration, as cells require pores >25 μm to migrate through materials.^70^ While these observed pore sizes are in the submicron range, larger pores could be achieved through processing modifications such as freeze-drying, where controlled freezing conditions can produce pore sizes ranging from micrometers to hundreds of micrometers by exploiting ice crystal formation during the freezing process, with pore size being dependent on cooling rate and temperature.^71,72^ The observed difference between conditions aligns with ionic cross-linking effects, in which ionic solutions produce more uniform pore distributions compared to deionized water processing.^72^ These structural variations likely contribute to the mechanical differences between SW–SW and DW–DW fibers.

## 5 Conclusions

This study characterizes the water content, swelling ratios, mechanical performance, and structural properties of recombinant hagfish intermediate filament (rHIF) protein hydrogel fibers. The water content of these rHIF hydrogel fibers (66–81% water by mass) are comparable to that of silk fibroin hydrogels while maintaining high mass and diameter swelling ratios (208–347%, and 81–215%, respectively). Further fine-tuning of the cross-linking levels or fiber diameter could further increase the water content of these hydrogels. The obtained mechanical results demonstrate that by varying protein concentration, composition, processing conditions, and environmental conditions, the stiffness (106–1041 kPa elastic modulus) of rHIF hydrogel fibers can be tuned to achieve a wide range of properties. This would allow for broad applications of these fibers, since they can mechanically match various cell types in an aqueous environment (i.e., 100 to 1200 kPa across several human tissues). FTIR-ATR analysis suggested a substantial increase in β-sheet content compared to the rHIF stretched, dry fibers, which was previously reported. However, AlphaFold3 (AF3) modeling predicted an intrinsically α-helical coiled-coil architecture, highlighting the need to interpret FTIR signals with caution, given the potential for misassignment of coiled-coils as β-sheets. The limitations of protein structure modeling should also be considered, since most systems rely on the protein sequence and assume the protein is in an aqueous environment, free from stresses present during fiber formation. SEM imaging further revealed nanoporous architectures spanning the fiber axis. Together, these findings establish rHIF hydrogel fibers as a versatile platform for designing next-generation protein-based biomaterials. Future studies to fine-tune the mechanical properties to targeted biomaterial applications could be done in combination with biocompatibility studies to further assess the potential of these rHIF hydrogel fibers in real-world biomedical applications.

## Supplementary Material



## Data Availability

The data underlying this study are available in the published article and its Supporting Information. Raw data used to generate the data presented in the article will be made available upon request.

## Notes

AlphaFold 3 (AF3) structure predictions for rHIF proteins; the top, side, and rear views of the 2 mm gap spacer used in the mechanical testing of the hydrogel fibers; and average fiber diameter measurements and calculations for the fibers examined in the water-content and swelling-ratio studies (PDF)
