# Supplementary material for: Tailorable Hydrogel Fibers from High-Yield Recombinant Hagfish Intermediate Filament Proteins: A New Frontier in Biomimetic Materials
Source: ACS Omega. 2026 May 30;11(23):33619–29. doi: 10.1021/acsomega.5c13031 (PMC13280683; doi:10.1021/acsomega.5c13031)
Supplement: Supplementary file 1 [file ao5c13031_si_001.pdf]

Supporting Information

Tailorable Hydrogel Fibers from High-Yield Recombinant Hagfish Intermediate Filament Proteins: A New Frontier in Biomimetic Materials

Brianne E. Bell[1‡], Oran Wasserman[1‡], Thomas I. Harris[2], Hayden B. Johns[1], Paula E. Oliveira[1], Justin A. Jones[1*]

[1]Department of Biology, Utah State University, Logan, UT 84322, USA.
[2] Naval Surface Warfare Center - Panama City Division, Panama City, FL 32407.
[‡]Authors contributed equally.
*Corresponding Author: Justin A. Jones. E-mail justin.a.jones@usu.edu; Tel.+1-435-797-9292.

9 Pages.

5 Figures.

1. AlphaFold3 structure predictions for rHIF proteins.
2. Drawing views of the 2 mm gap spacer used in mechanical testing.
3. Drawing views of the custom clamp device.
4. Correlation plots for the diameters, swelling ratios, and water content for 1:1 α:γ DW-DW and SW-DW.
5. Mechanical trends of the different proteins, concentrations, and the spinning and testing conditions.

3 Tables.

1. The protein type and concentration, and coagulation and test bath conditions for each test performed in this work.
2. Average diameter measurements and calculations for the fibers explored in the water content and swelling ratio studies
3. Full mechanical properties of the different proteins, concentrations, and the spinning and testing conditions.

AlphaFold3 was used to model the structures of the recombinant hagfish intermediate filament (rHIF) proteins alpha and gamma (α, γ). Monomeric, dimeric, and tetrameric structures were created for each protein type (Figure S1) to demonstrate protein structure and interactions.

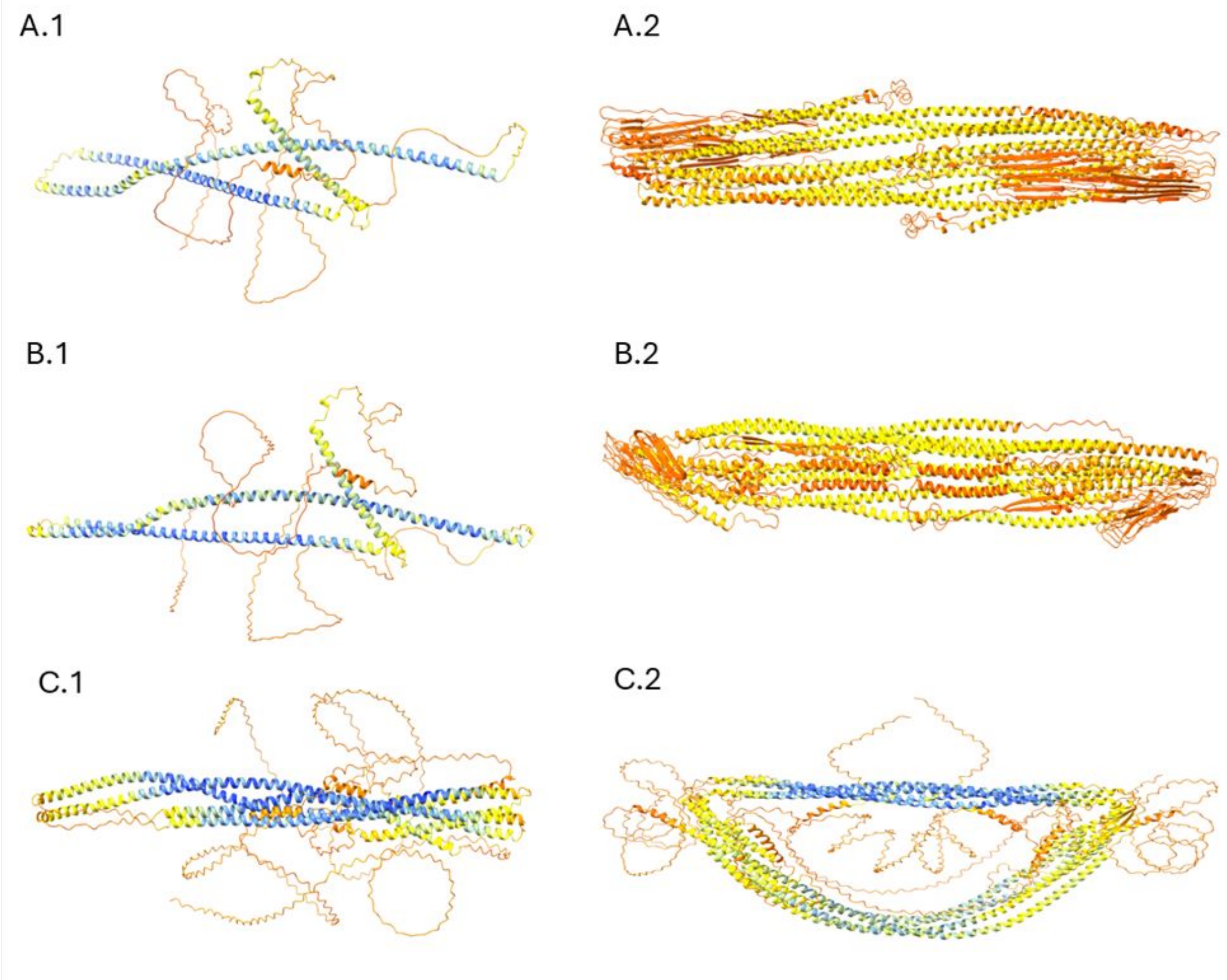

Figure S1. AlphaFold 3 (AF3) structure predictions for rHIF proteins. Predicted models of rHIF proteins where color gradients indicate predicted local distance difference test (pLDDT) scores: orange (0-50), yellow (50-70), cyan (70-90), and blue (90-100).[9] (A.1-A.2) α, (B.1-B.2) γ, and (C.1-C.2) 1:1 α:γ. (A.1, B.1) Monomeric structures, (C.1) Dimeric structures, and (A.2, B.2, C.2) tetrameric assemblies are displayed for each protein type.

Several tests were conducted on the rHIF hydrogel fibers in this study. These are outlined in Table S1, where each test is divided into the protein content and concentration, and the coagulation and testing bath solutions.

Table S1. Map of rHIF hydrogel fiber formulations, spinning and testing conditions selected for each experimental procedure.

**Test, Protein**	**% w/v**	**Coagulation-Test Water Bath Types**
Water Content and Mass Swelling Ratios		

α	-	-
1:1 α:γ	10, 15, 20, 25%	DW-DW, SW-DW
γ	-	-
Full Mechanical and Statistical Analysis (ANOVA)		
α	10, 15, 20, 25%	DW-DW, DW-SW, SW-DW, SW-SW
1:1 α:γ	10, 15, 20, 25%	DW-DW, DW-SW, SW-DW, SW-SW
γ	10, 15, 20, 25%	DW-DW, DW-SW, SW-DW, SW-SW
FTIR-ATR, Select Comparisons and Statistical Analysis (ANOVA)		
α	20%	SW-SW
	25%	DW-DW, SW-SW
1:1 α:γ	20%	SW-SW
	25%	DW-DW, SW-SW
γ	20%	SW-SW
	25%	DW-DW, SW-SW
SEM Imaging		
α	-	-
1:1 α:γ	20%	DW-DW, SW-SW
γ	-	-

Sketches generated from custom designs in SolidWorks Education Edition 2023 (Dassault Systèmes) depict the dimensions of the testing tower (Figure S2) and the custom clamping devices (Figure S3).

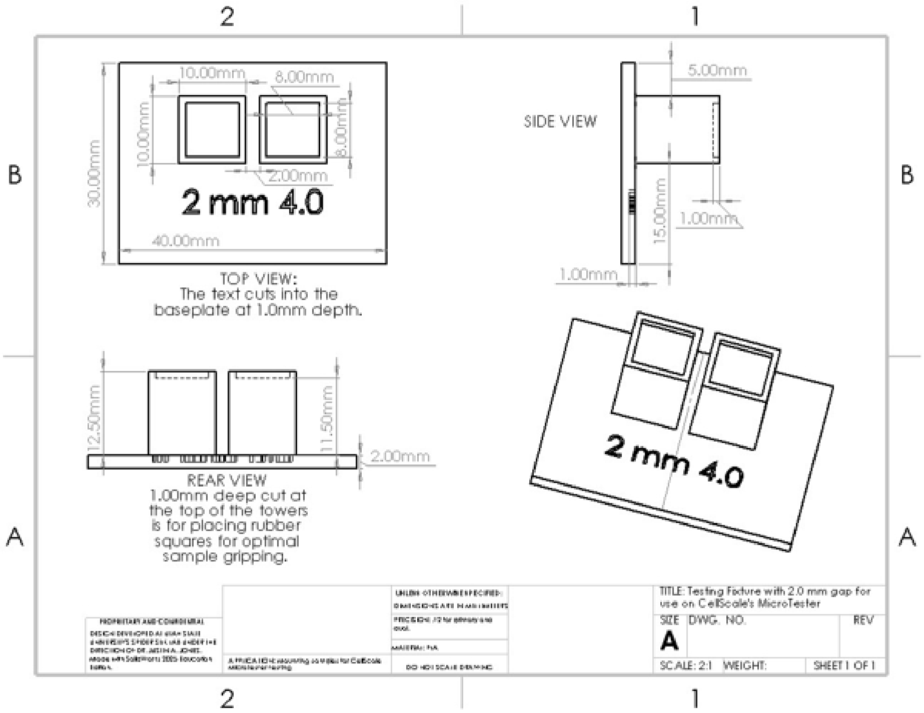

Figure S2. The top, side, and rear views of the 2 mm gap spacer used in the mechanical testing of the hydrogel fibers.

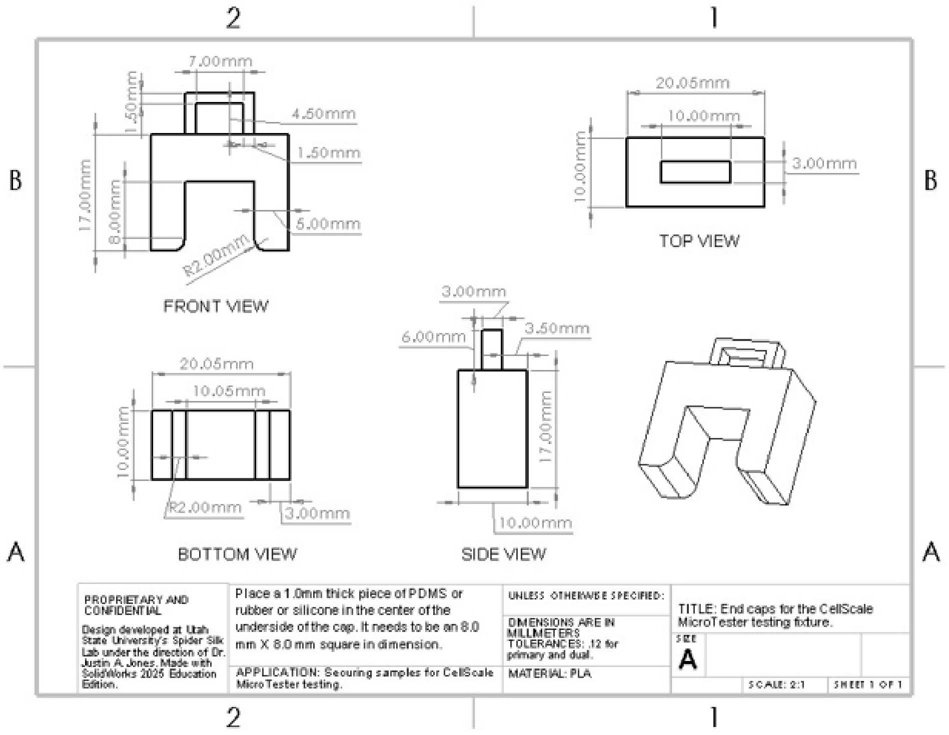

Figure S3. The front, top, side, and bottom views of the custom clamp device used in the mechanical testing of the hydrogel fibers. One clamp is to be placed on each vertical arm of the spacer device to fully secure the sample.

Table S2 contains the average values for both diameter types, water content, and the two swelling ratios calculated in the study. For these calculations and measurements, the 10-25% 1:1 α:γ DW-DW and SW-DW were used, which eliminated issues caused by salt from the SW-formed fibers influencing the wet mass measurements.

Table S2. Average fiber diameter measurements and calculations for the fibers examined in the water-content and swelling-ratio studies.

% w/v Concentration	Coagulation Water	D_{dry} (µm)	D_{wet} (µm)	Q_d (%)	Q_m (%)	WC (%)
10%	DW	36.2	114.2	215%	325%	81%
	SW	44.4	130.1	193%	347%	75%
15%	DW	57.7	132.9	130%	338%	78%
	SW	56.6	139.1	146%	282%	73%
20%	DW	73.9	137.6	86%	289%	75%
	SW	67.3	152.3	126%	208%	66%
25%	DW	79.3	143.1	81%	308%	75%
	SW	73.5	172.7	135%	233%	68%

Pearson correlation coefficients (Figure S4) for the lyophilized diameter (D_{dry}), hydrated diameter (D_{wet}), diameter swelling ratio (Q_d), mass swelling ratio (Q_m) and water content (WC) of the hydrogel fibers. Each plot used the data regardless of protein concentration, meaning that the DW and SW plots are the grouped correlation values for DW or SW, regardless of protein amount.

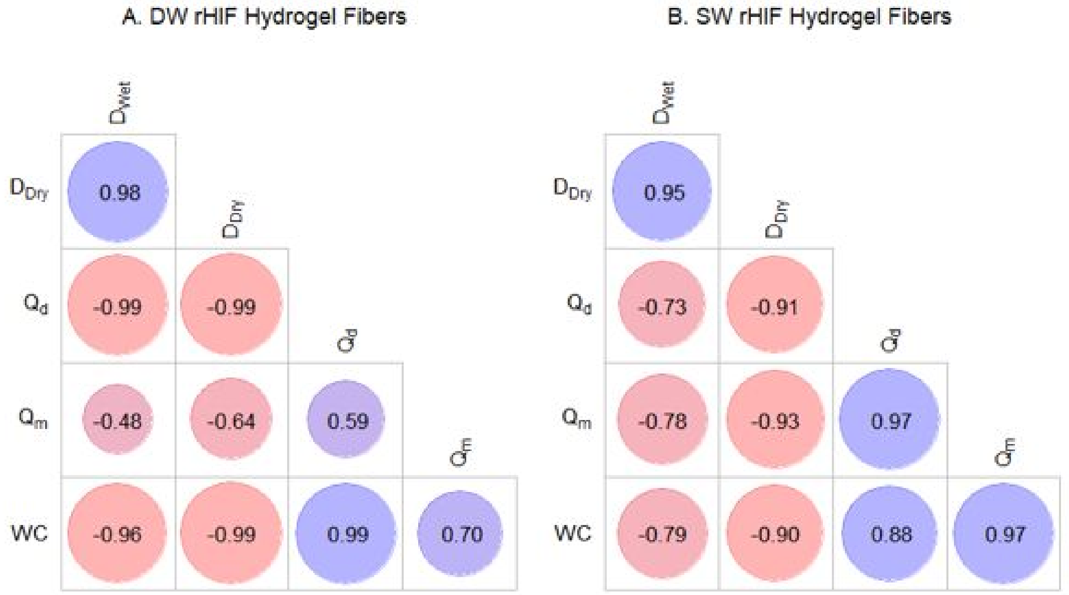

Figure S4. Correlation plots of the correlation coefficients relating to the diameter, swelling ratios, and water content of the hydrogel fibers. (A) fibers spun in DW, (B) fibers spun in SW but tested in DW.

Mechanical properties (mean ± standard deviation) and the overall ANOVA-Tukey analysis, with 95% confidence, of the hydrogel fibers at different protein concentrations and differing spinning and testing conditions are shown in Table S3.

Table S3. Full mechanical properties of the different proteins, concentrations, and the spinning and testing conditions. Sample size n is the number of tested fibers.

Spin-Test Baths	n	Stress (MPa)	Strain (mm mm^{-1})	Energy to Break (MJ m^{-3})	Elastic Modulus (kPa)	Diameter (micron)
10% w/v α						
DW-DW	11	0.34 ± 0.06 [H]	2.99 ± 0.31 [E-F]	0.48 ± 0.12 [G]	152.1 ± 55.3 [H-I]	120.1 ± 7.5 [F]
DW-SW	12	0.17 ± 0.06 [H]	3.35 ± 0.50 [D-F]	0.33 ± 0.11 [G]	105.9 ± 46.3 [I]	129.7 ± 9.6 [E-F]
SW-DW	11	0.38 ± 0.16 [G-H]	4.60 ± 1.84 [B-D]	1.02 ± 0.47 [F-G]	251.6 ± 76.6 [F-I]	128.7 ± 12 [E-F]
SW-SW	11	0.36 ± 0.10 [H]	4.92 ± 1.27 [B-C]	1.02 ± 0.23 [F-G]	206.7 ± 34.7 [G-I]	135.9 ± 4.7 [D-F]
15% w/v α						
DW-DW	11	0.78 ± 0.24 [F]	2.57 ± 0.22 [F]	0.92 ± 0.24 [F-G]	274.8 ± 88.9 [E-I]	145.1 ± 25 [C-E]
DW-SW	11	0.78 ± 0.20 [E-F]	4.64 ± 1.20 [B-D]	2.15 ± 1.26 [D-G]	302.8 ± 98.2 [E-H]	133.0 ± 14 [E-F]
SW-DW	10	1.96 ± 0.22 [A-B]	4.03 ± 0.28 [C-F]	3.84 ± 0.53 [B-D]	839.5 ± 75.6 [A]	136.2 ± 16 [C-F]
SW-SW	10	1.64 ± 0.42 [B-C]	4.83 ± 1.32 [B-D]	4.09 ± 1.42 [B-C]	611.2 ± 237 [B-C]	130.0 ± 8.5 [E-F]
20% w/v α						
DW-DW	10	1.03 ± 0.37 [E-F]	3.01 ± 0.32 [E-F]	1.55 ± 0.55 [F-G]	395.9 ± 88.7 [D-F]	154.2 ± 15 [B-D]
DW-SW	10	0.73 ± 0.21 [F-G]	6.07 ± 1.47 [A-B]	2.70 ± 0.67 [C-F]	319.9 ± 88.7 [E-H]	155.1 ± 16 [B-C]
SW-DW	10	1.15 ± 0.30 [D-E]	3.45 ± 0.35 [C-F]	2.09 ± 0.69 [D-G]	546.7 ± 164 [C-D]	174.7 ± 17 [A]
SW-SW	11	1.43 ± 0.21 [C-D]	5.99 ± 0.32 [A-B]	4.64 ± 0.45 [B]	436.8 ± 75.8 [C-E]	165.5 ± 5.7 [A-B]
25% w/v α						
DW-DW	15	1.04 ± 0.13 [E-F]	2.87 ± 0.23 [E-F]	1.47 ± 0.20 [F-G]	403.7 ± 130 [D-F]	178.5 ± 11 [A]
DW-SW	10	0.75 ± 0.14 [F]	4.27 ± 1.14 [C-E]	1.85 ± 0.69 [E-G]	370.2 ± 99.9 [E-G]	182.2 ± 8.3 [A]

SW-DW	11	1.99 ± 0.38 [A-B]	3.57 ± 0.28 [C-F]	3.64 ± 0.77 [B-E]	814.4 ± 216 [A]	173 ± 12.5 [A-B]
SW-SW	11	2.13 ± 0.39 [A]	7.35 ± 2.13 [A]	10.2 ± 4.16 [A]	763.5 ± 105 [A-B]	168.9 ± 6.2 [A-B]
10% w/v 1:1 α:γ						
DW-DW	11	0.53 ± 0.26 [E-H]	2.41 ± 0.32 [E]	0.57 ± 0.19 [F]	204.5 ± 82.7 [F]	114.2 ± 21 [H]
DW-SW	11	0.40 ± 0.07 [H]	5.69 ± 1.65 [A-B]	1.36 ± 0.50 [E-F]	177.5 ± 29.3 [F]	117.2 ± 6.4 [G-H]
SW-DW	12	0.48 ± 0.11 [F-H]	3.34 ± 0.23 [D-E]	0.91 ± 0.20 [E-F]	319.8 ± 76.7 [E-F]	130.1 ± 6.3 [E-H]
SW-SW	12	0.46 ± 0.08 [G-H]	4.15 ± 0.88 [C-D]	1.06 ± 0.38 [E-F]	225.4 ± 30.4 [F]	136.1 ± 13 [E-F]
15% w/v 1:1 α:γ						
DW-DW	20	0.93 ± 0.24 [D-E]	2.50 ± 0.24 [E]	1.14 ± 0.28 [E-F]	436.3 ± 97.7 [D-E]	132.9 ± 14 [E-G]
DW-SW	11	0.65 ± 0.18 [D-H]	4.39 ± 1.28 [B-D]	1.77 ± 1.03 [D-F]	262.6 ± 76.3 [E-F]	123.7 ± 12 [F-H]
SW-DW	10	1.05 ± 0.29 [B]	4.05 ± 0.54 [C-D]	2.28 ± 0.79 [B-E]	440.2 ± 109 [D-E]	139.1 ± 9.6 [D-F]
SW-SW	11	0.84 ± 0.23 [B-C]	5.79 ± 1.56 [A]	2.84 ± 1.33 [B-D]	271.0 ± 73.7 [E-F]	159.0 ± 11 [A-C]
20% w/v 1:1 α:γ						
DW-DW	12	1.06 ± 0.26 [C-D]	2.28 ± 0.22 [E]	1.24 ± 0.37 [E-F]	803.1 ± 259 [B-C]	137.6 ± 13 [D-F]
DW-SW	11	0.79 ± 0.16 [D-H]	3.90 ± 1.23 [C-D]	1.88 ± 0.98 [D-F]	377.0 ± 73.2 [E-F]	141.3 ± 15 [D-E]
SW-DW	12	1.67 ± 0.70 [B]	4.03 ± 0.57 [C-D]	3.44 ± 1.03 [B]	639.2 ± 277 [C-D]	152.3 ± 5.4 [B-D]
SW-SW	12	1.38 ± 0.33 [B-C]	4.42 ± 1.26 [B-D]	3.35 ± 1.81 [B-C]	589.5 ± 168 [D]	145.0 ± 7.7 [C-E]
25% w/v 1:1 α:γ						
DW-DW	12	1.60 ± 0.40 [B]	2.47 ± 0.23 [E]	2.01 ± 0.48 [C-E]	1041 ± 294 [A]	143.1 ± 13 [C-E]
DW-SW	12	0.88 ± 0.21 [D-F]	4.40 ± 1.27 [B-D]	2.29 ± 1.17 [B-E]	352.4 ± 51.6 [E-F]	167.6 ± 10 [A-B]
SW-DW	13	1.67 ± 0.19 [B]	3.83 ± 0.31 [C-D]	3.16 ± 0.51 [B-D]	616.4 ± 79.6 [C-D]	172.7 ± 6.0 [A]
SW-SW	14	2.24 ± 0.32 [A]	4.97 ± 1.00 [A-C]	6.20 ± 2.19 [A]	971.2 ± 102 [A-B]	153.3 ± 7.5 [B-D]
10% w/v γ						

DW-DW	11	0.29 ± 0.07 [F-G]	2.96 ± 0.19 [D-F]	0.45 ± 0.12 [F-G]	164.8 ± 56.5 [G-H]	124.3 ± 12 [D-E]
DW-SW	11	0.18 ± 0.07 [F-G]	3.39 ± 0.51 [B-F]	0.36 ± 0.14 [G]	142.1 ± 41.1 [G-H]	114.7 ± 6.8 [E]
SW-DW	11	0.18 ± 0.02 [F-G]	2.84 ± 0.24 [D-F]	0.33 ± 0.05 [G]	193.1 ± 40.4 [F-H]	134.2 ± 6.4 [C-E]
SW-SW	11	0.12 ± 0.03 [G]	4.10 ± 1.65 [B-D]	0.32 ± 0.12 [G]	115.1 ± 27.9 [H]	146.3 ± 4.7 [B-C]
15% w/v γ						
DW-DW	10	0.66 ± 0.20 [C-E]	2.48 ± 0.44 [F]	0.84 ± 0.26 [E-G]	318.1 ± 78.6 [D-F]	135.4 ± 16 [C-E]
DW-SW	10	0.43 ± 0.09 [E-F]	4.54 ± 1.88 [B-C]	1.14 ± 0.30 [D-F]	212.4 ± 72.7 [F-H]	133.5 ± 20 [C-E]
SW-DW	10	0.59 ± 0.16 [E]	3.30 ± 0.32 [C-F]	1.12 ± 0.25 [E-F]	365.6 ± 90.6 [C-E]	147.2 ± 12 [B-C]
SW-SW	12	0.58 ± 0.16 [E]	3.63 ± 0.67 [B-F]	1.23 ± 0.43 [D-E]	338.9 ± 43.6 [D-F]	140.7 ± 7.1 [C-D]
20% w/v γ						
DW-DW	11	0.91 ± 0.24 [B-C]	2.48 ± 0.23 [F]	1.11 ± 0.23 [E-F]	557.2 ± 300 [A-B]	147.2 ± 18 [B-C]
DW-SW	13	0.67 ± 0.30 [C-E]	6.00 ± 1.88 [A]	2.54 ± 1.32 [B]	313.9 ± 89.7 [D-F]	145.2 ± 22 [C-D]
SW-DW	11	1.11 ± 0.20 [A-B]	3.04 ± 0.26 [D-F]	1.83 ± 0.40 [C-D]	608.7 ± 62.7 [A]	167.3 ± 20 [A-B]
SW-SW	12	1.33 ± 0.30 [A]	4.73 ± 1.13 [A-B]	3.28 ± 0.48 [A]	502.9 ± 91.6 [A-C]	139.7 ± 11 [C-D]
25% w/v γ						
DW-DW	11	0.85 ± 0.13 [C-D]	2.74 ± 0.16 [E-F]	1.21 ± 0.18 [D-E]	382.9 ± 74.8 [C-E]	181.8 ± 12 [A]
DW-SW	11	0.63 ± 0.16 [D-E]	3.70 ± 0.41 [B-F]	1.3 ± 0.38 [D-E]	265.8 ± 87.3 [E-G]	186.7 ± 24 [A]
SW-DW	11	1.19 ± 0.15 [A]	3.47 ± 0.22 [B-F]	2.26 ± 0.33 [B-C]	531.2 ± 39.6 [A-B]	178.1 ± 3.4 [A]
SW-SW	11	1.18 ± 0.13 [A]	4.05 ± 0.41 [B-E]	2.54 ± 0.46 [B]	426.3 ± 49.4 [B-D]	179.2 ± 5.5 [A]

The overall mechanical trends of the hydrogel fibers at different protein concentrations and differing spinning and testing conditions are shown in Figure S5. The shorter dashed blue lines with circle markers correspond to the α hydrogel fibers. The purple solid lines with diamond markers represent the 1:1 α: γ hydrogel fibers. The orange dashed lines with triangle markers correspond to the γ hydrogel fibers.

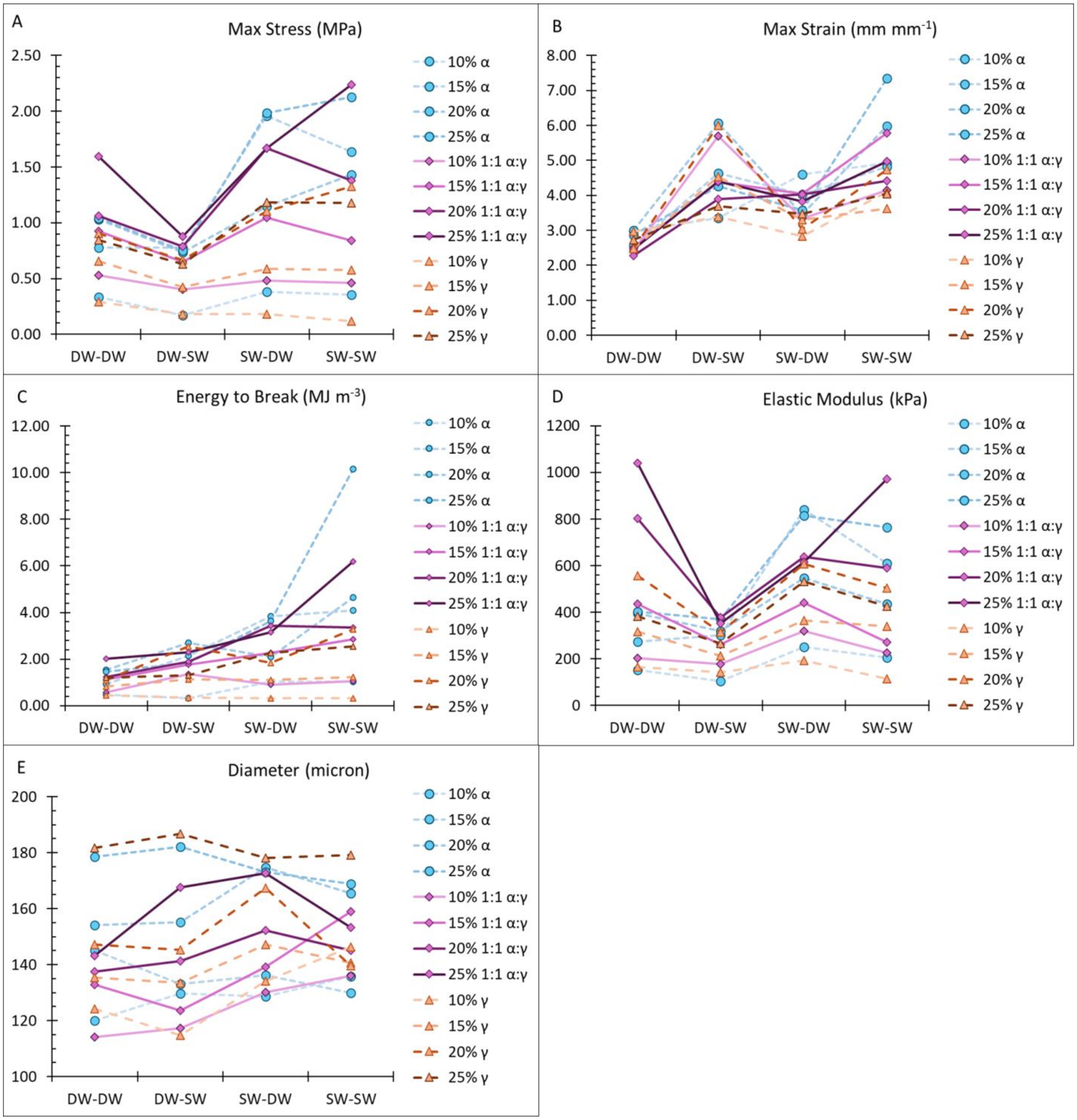

Figure S5. Mechanical trends of the different proteins, concentrations, and the spinning and testing conditions. (A) Stress (MPa), (B) Strain (mm mm^{-1}), (C) Energy to Break (MJ m^{-3}), (D) Elastic Modulus (kPa), and (E) Diameter (micron).
